# Y-chromosome evidence supports asymmetric dog introgression into eastern coyotes

**DOI:** 10.1002/ece3.693

**Published:** 2013-07-31

**Authors:** Tyler J Wheeldon, Linda Y Rutledge, Brent R Patterson, Bradley N White, Paul J Wilson

**Affiliations:** 1 Environmental and Life Sciences Graduate Program, Trent UniversityPeterborough, ON, Canada, K9J 7B8; 2 Natural Resources DNA Profiling and Forensics Center, Trent UniversityPeterborough, ON, Canada, K9J 7B8; 3 Ontario Ministry of Natural Resources, Trent UniversityPeterborough, ON, Canada, K9J 7B8

**Keywords:** Coyote, dog, haplotype, hybridization, introgression, wolf

## Abstract

Hybridization has played an important role in the evolutionary history of *Canis* species in eastern North America. Genetic evidence of coyote–dog hybridization based on mitochondrial DNA (mtDNA) is lacking compared to that based on autosomal markers. This discordance suggests dog introgression into coyotes has potentially been male biased, but this hypothesis has not been formally tested. Therefore, we investigated biparentally, maternally, and paternally inherited genetic markers in a sample of coyotes and dogs from southeastern Ontario to assess potential asymmetric dog introgression into coyotes. Analysis of autosomal microsatellite genotypes revealed minimal historical and contemporary admixture between coyotes and dogs. We observed only mutually exclusive mtDNA haplotypes in coyotes and dogs, but we observed Y-chromosome haplotypes (Y-haplotypes) in both historical and contemporary coyotes that were also common in dogs. Species-specific *Zfy* intron sequences of Y-haplotypes shared between coyotes and dogs confirmed their homology and indicated a putative origin from dogs. We compared Y-haplotypes observed in coyotes, wolves, and dogs profiled in multiple studies, and observed that the Y-haplotypes shared between coyotes and dogs were either absent or rare in North American wolves, present in eastern coyotes, but absent in western coyotes. We suggest the eastern coyote has experienced asymmetric genetic introgression from dogs, resulting from predominantly historical hybridization with male dogs and subsequent backcrossing of hybrid offspring with coyotes. We discuss the temporal and spatial dynamics of coyote–dog hybridization and the conditions that may have facilitated the introgression of dog Y-chromosomes into coyotes. Our findings clarify the evolutionary history of the eastern coyote.

## Introduction

The evolutionary histories and taxonomy of contemporary *Canis* species in North America are complex and controversial (see Nowak 2003; Chambers et al. 2012). There is agreement that the gray wolf (*C. lupus*) and coyote (*C. latrans*) are distinct species that evolved in Eurasia and North America, respectively (Nowak 1979; Kurtén and Anderson 1980), and that the dog (*C. familiaris*) was domesticated from the gray wolf (Wayne and Vilà 2003). The eastern wolf (*C. lycaon*) is taxonomically controversial (e.g., vonHoldt et al. 2011 vs. Rutledge et al. 2012a), but herein is considered an endemic North American species based on consideration of genetic and nongenetic data (Kyle et al. 2006; Mech 2011; Benson et al. 2012; Chambers et al. 2012). Hybridization has clearly played an important role in the evolutionary history of *Canis* species in eastern North America (see Wayne and Vilà 2003; Kyle et al. 2006; vonHoldt et al. 2011; Chambers et al. 2012).

Hybridization is an evolutionary process that can occur naturally or because of anthropogenic influences. Regional habitat change that facilitates range expansion of one species into the range of another can result in hybridization, potentially producing novel gene combinations that can allow for rapid evolutionary change (Rhymer and Simberloff 1996). Such was the case for the coyote, which hybridized with the eastern wolf as it expanded into northeastern North America (Parker 1995; Wilson et al. 2009; Kays et al. 2010).

Coyotes were historically confined to the western prairies and grasslands of North America (Young and Jackson 1951), until the eradication of wolves and landscape changes associated with European colonization facilitated coyote expansion eastward during the last century (Parker 1995). Coyotes differ noticeably in morphology and genetic composition between the western and eastern portion of the species’ range. The larger size of eastern coyotes (Way 2007) has been attributed to either a phenotypic response to enhanced food supply (Thurber and Peterson 1991) or a genotypic response to larger prey (Larivière and Crête 1993), the latter of which was suggested to have been facilitated by genetic variation introduced through hybridization with wolves (Kays et al. 2010). Eastern coyotes commonly exhibit introgressed wolf genes (Kays et al. 2010; Way et al. 2010; Bozarth et al. 2011; Wilson et al. 2012), which are absent in western coyotes (Lehman et al. 1991; Hailer and Leonard 2008; Koblmüller et al. 2009). The genetic evidence of wolf genes in eastern coyotes corroborates earlier suggestions, based on cranial measurements of “New England *Canis*”, that wolf genes had likely been introgressed into eastern coyotes (Lawrence and Bossert 1969; Mengel 1971). Furthermore, it was suggested that dog genes had likely been introgressed into eastern coyotes (Lawrence and Bossert 1969; but see Mengel 1971). Indeed, recent investigation of single-nucleotide polymorphisms (SNPs) has demonstrated dog admixture in eastern coyotes (vonHoldt et al. 2011), but it is intriguing that there is limited evidence of nonrecombining genes of dog origin in eastern coyotes, despite multiple studies investigating their genetic composition (Koblmüller et al. 2009; Kays et al. 2010; Way et al. 2010; Wheeldon et al. 2010a; Bozarth et al. 2011). We are aware of only the observations of a single-dog mitochondrial DNA (mtDNA) haplotype in coyotes in the southeastern United States (Adams et al. 2003) and one putative dog-like mtDNA sequence in a coyote in Vermont (Kays et al. 2010). Thus, there is an apparent disparity in the genetic data pertaining to coyote–dog hybridization, which may be attributable to the focus on mtDNA in previous studies, or insufficient comparisons of genetic data across *Canis* species to infer the origins of introgression. It is plausible that genetic introgression from dogs to eastern coyotes may have occurred more extensively through paternal contributions (e.g., Wheeldon and Patterson 2012), but this hypothesis has not been formally tested. Investigation of the Y-chromosome has proven valuable in assessing wolf–dog hybridization in Europe, revealing hybridization patterns that were not evident based on mtDNA (Iacolina et al. 2010; Godinho et al. 2011). Such could prove to be the case in assessing coyote–dog hybridization.

Investigations of the canine Y-chromosome have typically involved genotyping four to seven microsatellite loci that are combined into Y-haplotypes (e.g., Sundqvist et al. 2001; Fain et al. 2010), but the recent evaluation of diagnostic SNPs in the last intron of the *Zfy* gene has facilitated the inference of the species-specific origins of these Y-haplotypes (Wilson et al. 2012). The combined investigation of Y-chromosome SNPs and microsatellite alleles, which are highly conserved (i.e., ∼10^−8^ per site per generation in humans) and highly mutable (i.e., ∼10^−3^ per locus per generation in humans), respectively (see Hurles and Jobling 2001), facilitates the assessment of Y-haplotype diversity among species and the taxonomic origins of the genes. This approach is appropriate for the investigation of male-biased dog introgression into eastern coyotes because of anticipated multispecies introgression (vonHoldt et al. 2011; Wilson et al. 2012).

We generated autosomal microsatellite genotypes, mtDNA control region sequences, and Y-chromosome microsatellite genotypes and associated *Zfy* intron sequences for a sample of coyotes and dogs to investigate hybridization between these species in southeastern Ontario, Canada. The study region represents the putative geographic origin of the wolf–coyote hybridization that gave rise to the eastern coyote population that subsequently colonized the maritime Canadian provinces and northeastern United States (Hilton 1978; Parker 1995; Wheeldon et al. 2010a), therefore the coyotes sampled herein should be representative of the greater eastern coyote population. Specifically, we employed mtDNA and Y-chromosome markers to test the hypothesis that eastern coyotes have experienced male-biased genetic introgression from dogs, and we employed autosomal markers to assess levels of historical and contemporary gene flow between eastern coyotes and dogs. We note that our sampling of eastern coyotes is restricted to northeastern North America; reference to “eastern coyotes” with respect to our findings is intended to refer specifically northeastern coyotes.

## Material and Methods

### Samples and DNA extraction

**Figure 1:**
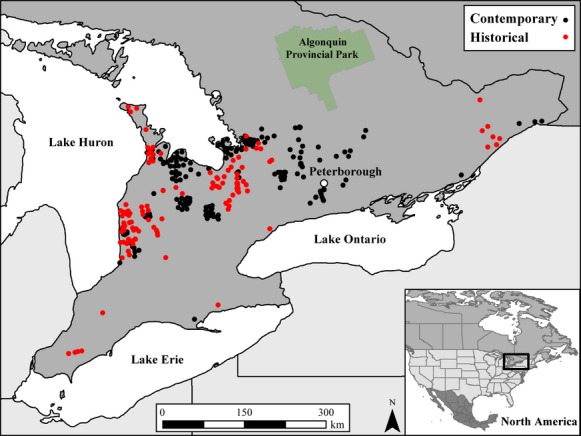
Approximate locations of historical (1974–1984) and contemporary (2005–2010) coyote samples collected from southeastern Ontario, Canada; some samples may be overlapping. The black square in the inset map depicts the location of the study area in North America. Dog samples were collected in the Peterborough region.

We obtained blood (*n* = 63), tissue (*n* = 273), and hair (*n* = 4) samples from 340 coyotes collected from southeastern Ontario during 1974–1984 (*n* = 120) and 2005–2010 (*n* = 220) (Fig. 1). The coyote samples were predominantly submitted by hunters and trappers or obtained from road kills. We generally restricted our sampling to south of 45° latitude, except for four samples collected east of 75° longitude and three collected west of 81° longitude (Fig. 1), to reduce the potential of sampling eastern wolves in the vicinity of Algonquin Provincial Park. We obtained tissue (*n* = 16) and blood (*n* = 59) samples of 75 domestic dogs collected during 2006–2011, which were obtained predominantly from a local veterinarian in the Peterborough region of Ontario (Fig. 1) and comprised mostly mixed-breed individuals. Breeds represented in the sampled dogs included Beagle, Border Collie, German Shepherd, Golden Retriever, Poodle, Husky, Labrador Retriever, Great Dane, Akita, Mastiff, Rottweiler, Malamute, Rhodesian Ridgeback, Spaniel, Terrier, and Hounds.

We extracted DNA from samples using a DNeasy Blood and Tissue Kit (Qiagen, Mississauga, ON, Canada), and determined gender by amplification of *Zfx*/*Sry* primer pairs (P1-5EZ and P2-3EZ: Aasen and Medrano 1990; Y53-3C and Y53-3D: Fain and LeMay 1995) or *Zfx*/*Zfy* primers (LGL-331 and LGL-335: Shaw et al. 2003).

### Autosomal and Y-chromosome microsatellite genotyping

For each sample we amplified 12 autosomal microsatellite loci in three multiplex reactions with published primers (cxx225, cxx2, cxx123, cxx377, cxx250, cxx204, cxx172, cxx109, cxx253, cxx442, cxx410, and cxx147: Ostrander et al. 1993, 1995) as in Wheeldon et al. (2010b). For male samples we amplified four Y-chromosome microsatellite loci with published primers (MS34A, MS34B, MS41A, and MS41B: Sundqvist et al. 2001), as in Wheeldon et al. (2010b). We purified amplified products through ethanol precipitation prior to genotyping on a MegaBACE 1000 (GE Healthcare, Baie d'Urfé, QC, Canada) or an AB3730 (Applied Biosystems, Burlington, ON, Canada). We accounted for allele shifts between instruments with multiple control samples and scored alleles in Genemarker (v1.7, Softgenetics LLC, State College, PA).

### Mitochondrial DNA control region and Z*fy* intron amplification and sequencing

We amplified a 343–347 base pair (bp) fragment of the control region of the mtDNA with published primers (AB13279: Pilgrim et al. 1998; AB13280: Wilson et al. 2000) as in Wheeldon et al. (2010b). For some samples, we amplified the same fragment of the mtDNA control region with different published primers (ThrL, DL-Hcan: Leonard et al. 2002) under similar conditions to those in Wheeldon et al. (2010b). For a subsample of males we amplified a 658 bp fragment of the last intron of the *Zfy* gene with published primers (LGL-331: Shaw et al. 2003; Yint-2-335: Wilson et al. 2012) as in Wilson et al. (2012). We purified polymerase chain reaction (PCR) products using Exosap-IT (USB Corporation, Cleveland, OH), or Exonuclease I and Antarctic Phosphatase (New England BioLabs Inc., Ipswich, MA), prior to sequencing on a MegaBACE 1000 (GE Healthcare) or an AB3730 (Applied Biosystems). We edited and aligned sequences in Bioedit (v7.0.9, Hall 1999) or MEGA (v5, Tamura et al. 2011). Many of the coyote samples analyzed herein were previously analyzed at the mtDNA control region by Wheeldon et al. (2010a).

### Genetic data analysis

We obtained 411 autosomal microsatellite genotypes based on 12 loci for the coyote and dog samples; four coyote samples (*n* = 3 historical; *n* = 1 contemporary) that amplified at less than six loci were excluded from subsequent analyses. Furthermore, we obtained genotypes of genetically assigned eastern wolves from Algonquin Provincial Park (*n* = 62: Rutledge et al. 2010) and gray wolves from northeastern Ontario (*n* = 62: Holloway 2009; Rutledge et al. 2010; Wheeldon and Patterson 2012) for inclusion in autosomal data analyses to account for possible admixture from these groups in the sampled coyotes and to identify potential noncoyote migrants sampled in southeastern Ontario. We acknowledge the mixed ancestry of these wolf groups, but we refer to them as eastern wolves and gray wolves for simplicity.

We analyzed the individual autosomal microsatellite genotypes in the Bayesian-clustering program Structure (v2.3, Pritchard et al. 2000; Hubisz et al. 2009) to assess admixture between coyotes and dogs. For all Structure analyses we inferred the parameter alpha and implemented the F-model (assumes correlated allele frequencies) and I-model (assumes independent allele frequencies) separately to compare results. We analyzed the historical and contemporary coyotes separately to avoid clustering problems associated with disparate sample sizes among groups. Based on prior findings (Rutledge et al. 2010; Benson et al. 2012) and accounting for the inclusion of dogs, we anticipated that *K* = 4 would be optimal given the data. To confirm this for the data set that included contemporary coyotes, eastern wolves, gray wolves, and dogs, we ran the admixture model of Structure for *K* = 1–7 with five repetitions of 10^6^ iterations following a burn-in period of 10^5^ iterations for each *K*. We calculated the mean posterior probability (ln *P*[*D*]) for each *K* by averaging across the five runs and confirmed that *K* = 4 was optimal for the data set based on quantitative criteria (Fig. S1; ln *P*[*D*], Pritchard et al. 2000; Δ*K*, Evanno et al. 2005) and consideration of the biological significance of clusters. Additionally, we performed a factorial correspondence analysis on the entire data set of individual autosomal microsatellite genotypes (*n* = 535) as implemented in GENETIX (Belkhir et al. 2004) and observed clustering patterns generally concordant with the results from Structure (Fig. S2). Notably, there was complete overlap of the historical and contemporary coyote samples (Fig. S2), indicating that *K*-determination in Structure was justifiably unnecessary for the data set that included historical coyotes. Subsequently, for both data sets, we ran the admixture model of Structure 10 times at *K* = 4 for 10^6^ iterations following a burn-in period of 10^5^ iterations and collected information on the 90% probability intervals of individual assignments (ANCESTDIST = 1). We obtained individual admixture proportions (*Q*-values) from the run with the highest posterior probability and lowest variance; we considered individuals to be admixed, if *Q* < 0.8 (e.g., Vähä and Primmer 2006). We compared individual assignments between the F-model and I-model and observed general concordance (Fig. S3). Specifically, we observed five cases of an individual being assigned as admixed under the F-model but not admixed under the I-model model, and five cases of the reverse scenario (Fig. S3). We suggest neither model was optimal for our data set considering the variable evolutionary relationships between *Canis* species, therefore we averaged *Q*-values between the F-model and I-model; hereafter references to individual assignments are based on the averaged *Q*-values, unless stated otherwise.

We generated 223–228 bp mtDNA sequences and distinguished those evolved in gray wolves and dogs (i.e., Old World origin) from those evolved in coyotes and eastern wolves (i.e., New World origin) based on a diagnostic indel (Pilgrim et al. 1998; Wilson et al. 2000).

We generated 400 bp *Zfy* intron sequences and observed three previously described sequences based on two variable sites: Yint-1 of coyote origin; Yint-2 of gray wolf (or dog) origin; and Yint-4 of eastern wolf origin (Wilson et al. 2012). We did not observe Yint-3 of coyote origin (Wilson et al. 2012) in our sample, which probably reflects a founder effect whereby eastward colonizing coyotes carried the Yint-1 sequence.

We generated Y-haplotypes based on the Y-chromosome microsatellite genotypes. We constructed a median-joining network (Bandelt et al. 1999) based on combined Y-chromosome microsatellite and *Zfy* intron data using the program Network (v4.6.1.0; available at http://www.fluxus-engineering.com/sharenet.htm) with default settings (ε = 0). The Y-chromosome microsatellite loci were weighted equally and the *Zfy* intron sequence variation was weighted twice as high as microsatellite loci.

We compared the Y-haplotypes that we observed in coyotes and dogs in this study with those of previously analyzed wolves, coyotes, and dogs to investigate Y-haplotype sharing among these species for the purpose of inferring introgression. We defined western and eastern coyotes as occurring west and east of the Mississippi River, respectively, and Great Lakes coyotes as occurring in the western Great Lakes states and western Ontario (Wheeldon et al. 2010b). We defined Great Lakes wolves as occurring in Manitoba, northern Ontario, southern Quebec, and the western Great Lakes states (Wheeldon 2009). Allele sizes for this study were calibrated with those in Rutledge et al. (2010), Wheeldon et al. (2010b), Wheeldon and Patterson (2012), and Wilson et al. (2012), the last of which calibrated allele sizes with those in Hailer and Leonard (2008). Furthermore, allele sizes were standardized among Musiani et al. (2007), Hailer and Leonard (2008), Sundqvist et al. (2001, 2006) (allele sizes obtained from the authors), and Fain et al. (2010).^1^ Thus, allele sizes were standardized for comparisons across studies. We generated unique Y-haplotype identifiers (Yh*n*) for each Y-chromosome microsatellite genotype because existing identifiers were not the same for matching Y-haplotypes across studies (Table S1). We performed a reduced-median analysis (*r* = 2; Bandelt et al. 1995) of the Y-haplotypes (loci weighted equally; frequency >1) and then constructed a median-joining network (ε = 10) with maximum parsimony postprocessing (Polzin and Daneshmand 2003).

## Results

### Autosomal microsatellite data

**Figure 2:**
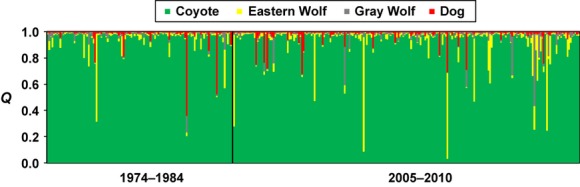
Structure assignments of historical (1974–1984) and contemporary (2005–2010) coyotes from southeastern Ontario genotyped at 12 autosomal microsatellite loci. Each partitioned vertical bar represents an individual's proportional membership to the *K* = 4 populations analyzed in Structure; *Q*-values represent the average of the F-model and I-model results.

We observed in coyotes varying levels of autosomal admixture from dogs, eastern wolves, and/or gray wolves (Fig. 2). One contemporary coyote was identified as an eastern wolf migrant^2^ (*Q*_EW_ > 0.8) and another was identified as eastern wolf × dog admixed with negligible coyote assignment (Fig. 2); both individuals were omitted from further consideration. Approximately 94% of historical (*n* = 110) and 89% of contemporary (*n* = 194) coyotes had individual assignments with *Q*_COY_ ≥ 0.8, and ∼3% of historical (*n* = 3) and contemporary (*n* = 6) coyotes had individual assignments with *Q*_DOG_ > 0.2 (Fig. 2). Approximately 2% of historical (*n* = 2) and 4% of contemporary (*n* = 9) coyotes had individual assignments with *Q*_EW_ > 0.2, and no historical (*n* = 0) and 1% of contemporary (*n* = 3) coyotes had individual assignments with *Q*_GW_ > 0.2 (Fig. 2). All the historical and contemporary coyotes with *Q*_COY_ ≥ 0.8 had 90% probability intervals for *Q*_COY_ that did not overlap zero (both models), whereas only two historical coyotes (both models) and one contemporary coyote (F-model only) with *Q*_DOG_ > 0.2 had 90% probability intervals for *Q*_DOG_ that did not overlap zero (Table S2). Autosomal genotypes of several coyotes that had individual assignments with *Q*_DOG_ > 0.2 exhibited 1–2 alleles that were relatively common in dogs but rare in coyotes, corroborating that admixture had occurred.

### Mitochondrial DNA sequence data

We observed 11 and 15 mutually exclusive mtDNA haplotypes in coyotes and dogs, respectively (Table S3). The mtDNA haplotypes observed in dogs were exclusively of Old World origin (i.e., dog or gray wolf) and those observed in coyotes were exclusively of New World origin (i.e., coyote or eastern wolf).

### Y-chromosome data

**Figure 3:**
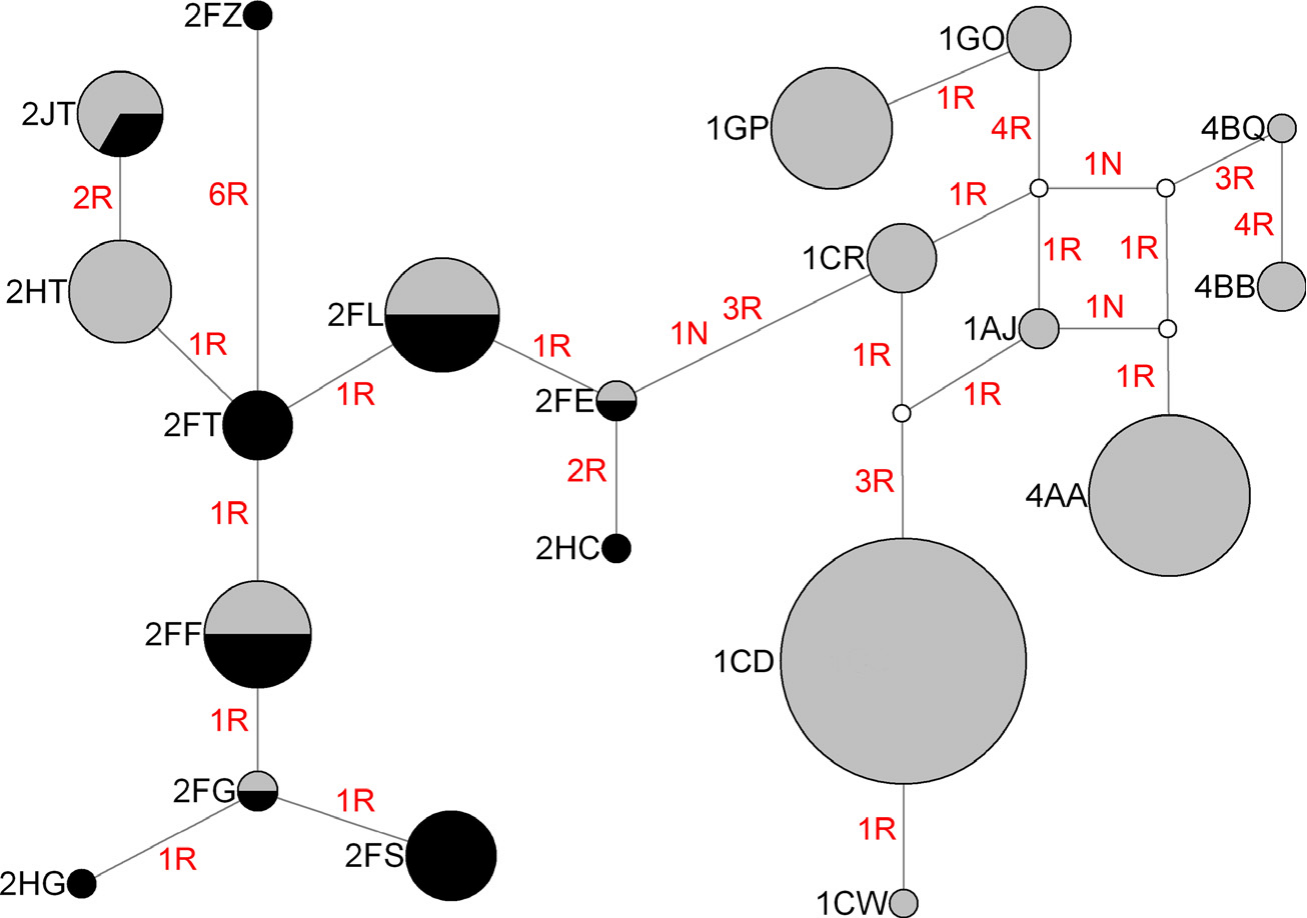
Median-joining network of Y-chromosome haplotypes observed in coyotes from southeastern Ontario and dogs. Haplotypes are composites of Y-chromosome microsatellite genotypes and *Zfy* intron sequences and are represented by nodes that have sizes proportional to their frequency: gray, coyote; black, dog. Median vectors are represented by white nodes. Red text on branch lengths indicates the number of mutational steps between nodes: N, nucleotide substitution; R, microsatellite repeat.

We obtained Y-chromosome microsatellite genotypes for 218 male samples and observed 20 Y-haplotypes. Specifically, we observed 15 and 10 Y-haplotypes in coyotes and dogs, respectively; five were shared between coyotes and dogs (Table 1). We obtained *Zfy* intron sequences for all Y-haplotypes and found that those observed exclusively in coyotes were associated with Yint-1 (*n* = 6), Yint-2 (*n* = 1), or Yint-4 (*n* = 3), and those observed exclusively in dogs were associated exclusively with Yint-2 (Table 1). The Y-haplotypes shared between coyotes and dogs were associated exclusively with Yint-2 (Table 1). The Y-haplotype network revealed a clear separation between the coyote-specific and dog-specific Y-haplotypes (Fig. 3); we note that Y-haplotype HT has been observed in dogs (Table 2), therefore we consider it shared regardless of it not being observed in dogs profiled in this study. The Y-haplotypes shared between coyotes and dogs clustered with the dog-specific Y-haplotypes (Fig. 3), therefore we consider them as belonging to the dog Y-haplogroup. Accordingly, there was a minimum of three microsatellite mutational steps and a *Zfy* intron substitution between the coyote and dog Y-haplogroups (Fig. 3).

**Table 1:** Y-chromosome haplotypes and associated *Zfy* intron sequences observed in historical (1974–1984) and contemporary (2005–2010) coyotes from southeastern Ontario and dogs. Haplotype frequencies are provided and the number of samples that had the *Zfy* intron sequenced for each haplotype is indicated in parentheses. Haplotypes are grouped by *Zfy* intron and sorted based on the allele at locus MS41A

Haplotype	MS34A	MS34B	MS41A	MS41B	*Zfy* intron	Historical coyotes	Contemporary coyotes	Dogs
CD	172	178	214	210	1	28 (0)	47 (6)	
CW	172	178	214	208	1		1 (1)	
AJ	172	180	212	214	1		2 (2)	
CR	172	178	212	216	1	4 (2)	2 (2)	
GO	176	180	212	220	1	1 (1)	4 (2)	
GP	176	180	212	222	1	7 (1)	11 (2)	
BB	170	182	212	226	4	1 (1)	2 (1)	
BQ	170	182	212	218	4		1 (1)	
AA	172	180	212	212	4	13 (1)	19 (1)	
FE	174	178	208	216	2	1 (1)		1 (1)
FF	174	178	208	222	2		7 (3)	7 (3)
FG	174	178	208	224	2		1 (1)	1 (1)
FL	174	178	208	218	2	2 (2)	6 (2)	8 (3)
FS	174	178	208	226	2			10 (3)
FT	174	178	208	220	2			6 (3)
HC	174	176	208	214	2			1 (1)
HG	174	176	208	224	2			1 (1)
HT	174	176	208	220	2		13 (3)	
JT	174	172	208	220	2	1 (1)	5 (3)	3 (3)
FZ	174	178	196	220	2			1 (1)

**Table 2:** Y-chromosome haplotypes and associated *Zfy* intron sequences observed in coyotes, wolves, and dogs; frequencies are provided (some may be overestimated due to the possibility of duplicate samples between studies). Microsatellite allele sizes were standardized across studies. Haplotypes are grouped by *Zfy* intron and sorted based on the allele at locus MS41A

Haplotype	*Zfy* intron	MS34A	MS34B	MS41A	MS41B	WC^1^^,^^2^	GLC^3^^,^^4^	EC^2^^,^^5^^,^^6^^,^^7^	EW^6^	GLW^2^^,^^3^^,^^4^^,^^6^^,^^7^	NA GW^1^^,^^2^^,^^3^^,^^8^^,^^9^	EA GW^10^	DD^5^^,^^8^
Yh1	1^11^	172	178	216	220		1						
Yh2 (CD)	1	172	178	214	210		3	107					
Yh3	1^2^	172	178	214	214	14	8	1					
Yh4	1^2^	172	178	214	216	7	4						
Yh5	1^2^	172	178	214	218	8	10	9					
Yh6	1^12^	172	178	214	212	1	6						
Yh7	1^12^	172	178	214	222		4						
Yh8 (CW)	1	172	178	214	208			1					
Yh9 (AJ)	1	172	180	212	214	2	7	2					
Yh10	1^2^	172	180	212	218	4	2						
Yh11	1^11^	172	180	212	216	3	4						
Yh12	1^2^	172	178	212	224	7							
Yh13	1^2^	172	178	212	220	3	1						
Yh14	1^2^	172	178	212	222	4							
Yh15	1^2^	172	178	212	218	4	2						
Yh16 (CR)	1	172	178	212	216		1	6					
Yh17	1^2^	172	176	212	218	4	3						
Yh18 (GO)	1	176	180	212	220			5					
Yh19 (GP)	1	176	180	212	222		1	28	1				
Yh20	3^2^	174	176	214	214	1							
Yh21	3^2^	174	178	212	212	2							
Yh22	3^2^	174	176	212	224	11							
Yh23	3^2^	174	176	212	220	7							
Yh24	3^2^	174	176	212	222	2							
Yh25	3^2^	174	180	212	218	1							
Yh26	3^2^	176	178	212	212	6							
Yh27	3^2^	176	178	212	214	1	1						
Yh28	3^2^	176	178	212	220	3							
Yh29	3^2^	176	178	208	214	2							
Yh30		172	176	218	214	1							
Yh31		172	178	216	210	3							
Yh32		172	180	214	214		1						
Yh33		172	178	214	220	2							
Yh34		172	178	214	224	2							
Yh35		174	178	214	216	1	1						
Yh36		172	178	212	214		7						
Yh37		174	176	212	226	1							
Yh38		174	180	212	214	1							
Yh39		174	174	212	220	4							
Yh40		174	176	212	214	1							
Yh41		176	178	212	210	3							
Yh42		176	178	212	222	1							
Yh43		178	176	212	220	3							
Yh44 (BB)	4	170	182	212	226			6	12	118			
Yh45	4^11^	170	182	212	222		9						
Yh46 (BQ)	4	170	182	212	218			1					
Yh47 (AA)	4	172	180	212	212		1	71	22	18			
Yh48	4^11^	172	182	212	212		2						
Yh49		170	176	210	220								1
Yh50		176	178	210	224							5	
Yh51	2^2^	172	180	208	222					31	41		
Yh52	2^11^	172	180	208	224					4	1		
Yh53	2^2^	172	180	208	220					1	3		
Yh54	2^2^	172	178	208	214					20	33		
Yh55	2^2^	172	178	208	216			2		57	32		
Yh56	2^2^	172	178	208	222			1		10	18		
Yh57	2^2^	172	178	208	224				1	1	37		
Yh58	2^2^	172	178	208	226				1	34	25		
Yh59	2^2^	172	178	208	220					15	27		
Yh60	2^2^	172	176	208	214					7	53		
Yh61 (FE)	2	174	178	208	216			1					2
Yh62 (FF)	2	174	178	208	222			25				4	64
Yh63 (FG)	2	174	178	208	224		4	6		2			16
Yh64 (FL)	2	174	178	208	218		1	17					106
Yh65 (FS)	2	174	178	208	226							4	13
Yh66 (FT)	2	174	178	208	220					1	10^13^		36
Yh67 (HC)	2	174	176	208	214								1
Yh68 (HG)	2	174	176	208	224								17
Yh69	2^2^	174	176	208	226			1					5
Yh70 (HT)	2	174	176	208	220		1	16				1	19
Yh71 (JT)	2	174	172	208	220			6					7
Yh72	2^2^	176	178	208	222						7	4	6
Yh73 (FZ)	2	174	178	196	220								10
Yh74		172	178	208	218						6^13^		
Yh75		172	176	208	226						9		
Yh76		172	180	208	214						2	4	
Yh77		172	178	208	212		1				3		
Yh78		172	176	208	216						1		
Yh79		172	180	208	226						2		
Yh80		172	178	208	228						1		
Yh81		172	182	208	214							3	
Yh82		172	182	208	220							10	
Yh83		172	180	208	216							13	
Yh84		172	180	208	212							1	
Yh85		172	182	208	216							1	
Yh86		172	184	208	222						2		
Yh87		174	178	208	228							8	
Yh88		174	178	208	214							1	4
Yh89		174	180	208	224								1
Yh90		174	176	208	222								7
Yh91		174	176	208	216								2
Yh92		174	182	208	220								5
Yh93		174	178	208	212								2
Yh94		174	184	208	216								1
Yh95		174	174	208	220								1
Yh96		174	180	208	218								1
Yh97		176	178	208	220						3		
Yh98		176	176	208	214						1		
Yh99		176	180	208	222						1		
Yh100		176	176	208	218							8	
Yh101		176	178	208	218								2
Yh102		176	184	208	218								1
Yh103		176	182	208	224								1
Yh104		178	176	208	218						1	15	1
Yh105		178	176	208	216							17	
Yh106		178	174	208	216							1	
Yh107		178	176	208	220								10
Yh108		178	178	208	220								1
Yh109		180	174	208	228								6

Corresponding haplotype names from Table 1 are indicated in parentheses where applicable. Sample group abbreviations: WC, western coyotes; GLC, Great Lakes coyotes; EC, eastern coyotes; EW, eastern wolves; GLW, Great Lakes wolves; NA GW, North American gray wolves (includes Mexican wolves); EA GW, Eurasian gray wolves; DD, domestic dogs. Geographic sampling information is provided as provincial or state abbreviations in the table footnotes: CBP, captive breeding program.

1 Data from Hailer and Leonard (2008): WC: TX, NE; NA GW: Mexican wolf CBP.

2 Data from Wilson et al. (2012): WC: TX, SK; EC: ME, NY, NB; GLW: QC, MB; NA GW: NT.

3 Data from Fain et al. (2010): GLC: WI; GLW: MN, MI, WI; NA GW: AK, BC, AB.

4 Data from Wheeldon et al. (2010b): GLC and GLW (*Q* ≥ 0.8 in Structure): MN, MI, WI, ON.

5 Data from this study: EC: ON; DD: ON.

6 Data from Rutledge et al. (2010): EC: ON; EW (*Q* ≥ 0.8 in Structure): ON; GLW: ON.

7 Data from Wheeldon and Patterson (2012): EC and GLW (*Q* ≥ 0.8 in Structure): ON.

8 Data from Sundqvist et al. (2006): NA GW: NT, AK; DD: unknown.

9 Data from Musiani et al. (2007): NA GW: NT, AK.

10 Data from Sundqvist et al. (2001): Scandinavia, Swedish Zoo, Finland, Baltic States, Russia.

11 Data from Wheeldon (unpublished data).

12 Data from Wheeldon (2009).

13 Data from Mexican wolves.

Comparison of the Y-haplotypes observed in coyotes and dogs in this study with those observed in wolves, coyotes, and dogs in previous studies revealed Y-haplotype sharing variously between and among species (Table 2). We observed Y-haplotypes in coyotes that were shared with dogs (*n* = 7), eastern wolves (*n* = 3), Great Lakes wolves (*n* = 5), North American gray wolves (*n* = 3), and Eurasian gray wolves (*n* = 2); some of these Y-haplotypes were shared jointly with two wolf groups, or with wolves and dogs. Eastern coyotes shared more Y-haplotypes with dogs (*n* = 7) than did Great Lakes coyotes (*n* = 3) or western coyotes (*n* = 0). We also observed Y-haplotypes that were shared exclusively between and among wolf groups, or between wolves and dogs.

## Discussion

### Autosomal admixture

We observed a low proportion (3%) of coyotes exhibiting autosomal admixture from dogs (Fig. 2), and the proportion was the same during the historical and contemporary sampling periods, suggesting that coyote–dog hybridization has been minimal during the past 35–40 years in southeastern Ontario. We observed a low proportion (4%) of coyotes exhibiting autosomal admixture from eastern wolves or gray wolves (Fig. 2), concordant with our sampling of coyotes outside of primary wolf range in Ontario; some of the coyotes that exhibited autosomal admixture from wolves could have been dispersing individuals from central Ontario where contemporary wolf–coyote hybridization occurs (Rutledge et al. 2010; Benson et al. 2012). The proportion of coyotes exhibiting autosomal admixture from eastern wolves or gray wolves was higher in the contemporary group (5%) than the historical group (2%), which likely reflects geographical differences in sampling between time periods, rather than a change in the frequency of wolf–coyote hybridization, because there were many contemporary but few historical samples collected from locations near where wolves occurred (Fig. 1; Benson et al. 2012).

### Introgression of nonrecombining markers

We observed mtDNA haplotypes of coyote or eastern wolf origin, but not those of dog or gray wolf origin, in our sample of coyotes (Table S3), which is concordant with previous studies (e.g., Koblmüller et al. 2009; Way et al. 2010; Bozarth et al. 2011; but see Adams et al. 2003; Kays et al. 2010). Notably, mtDNA haplotype C13 was only observed in one individual, which assigned predominantly as eastern wolf in Structure (Table S2), consistent with the lack of observation of this coyote-clustering sequence in coyotes and it being part of the eastern wolf lineage (Wheeldon and White 2009; Fain et al. 2010).

We observed Y-haplotype sharing between dogs and coyotes (Table 2), which we attribute to introgression from dogs into coyotes. We reject homoplasy as a potential cause of the observed Y-haplotype sharing between dogs and coyotes because the *Zfy* intron sequences of the shared Y-haplotypes confirm their homology and putative origin from dogs (Tables 2; Wilson et al. 2012). Furthermore, multiple mutational steps would need to have occurred in coyote Y-chromosomes for homoplasy to have generated the Y-haplotypes shared with dogs (Fig. 3), which seems unlikely. We reject the hypothesis that the observed Y-haplotype sharing between coyotes and dogs is attributable to a shared common ancestor given that they are present in eastern coyotes but apparently absent in western coyotes (Table 2), the latter of which represent the source of eastward colonizers that evolved into the former; this implies that coyotes experienced genetic introgression during eastward range expansion. Similarly, the eastern-specific presence in coyotes of the Y-halpotypes shared between coyotes and gray wolves or eastern wolves implies that they occur in coyotes due to introgression (Wilson et al. 2012); probably derived via hybridization with eastern wolves (Rutledge et al. 2010).

The Y-haplotype sharing that we observed between wolves and dogs (Table 2) could variously be the result of shared ancestry, homoplasy (e.g., Fig. S4; Sundqvist et al. 2006; Hailer and Leonard 2008), historical hybridization (Vilà et al. 1997), or contemporary hybridization (e.g., Godinho et al. 2011; Hindrikson et al. 2012), but formal assessment of these alternatives is beyond the scope of this study. Despite sharing multiple Y-haplotypes, wolves and dogs also exhibited multiple mutually exclusive Y-haplotypes (Table 2). Notably, of the seven Y-haplotypes shared between coyotes and dogs, none were observed in North American gray wolves and eastern wolves, one was observed in Great Lakes wolves, and two were observed in Eurasian gray wolves (Table 2), the last of which are not a plausible source of introgression for coyotes because of geographical considerations. The contemporary haplotype diversities of North American wolves (i.e., gray wolves, Great Lakes wolves, and eastern wolves) generally do not reflect their historical haplotype diversities because of genetic drift associated with drastic changes in population sizes (Leonard et al. 2005; Leonard and Wayne 2008). Accordingly, more of the Y-haplotypes shared between coyotes and dogs may have occurred in North American wolves historically, such that potentially these Y-haplotypes could have been introgressed into coyotes from wolves historically. However, gray wolves are aggressive toward and kill coyotes (e.g., Thurber et al. 1992; Berger and Gese 2007; Merkle et al. 2009), and genetic data indicate that these species do not interbreed in the wild (e.g., Pilgrim et al. 1998; Koblmüller et al. 2009); we are not aware of any successful interbreeding between these species in captivity. Similarly, Great Lakes wolves are aggressive toward and kill coyotes (see Mech 2011) and rarely hybridize with them (e.g., Fain et al. 2010; Wheeldon et al. 2010bb). Eastern wolves readily hybridize with coyotes (Rutledge et al. 2010) and therefore potentially could have mediated gene flow from dogs to coyotes historically, but there is no evidence of dog introgression in eastern wolves sampled to date (Table 2; Rutledge et al. 2012b; Benson et al. 2012). Coyotes and dogs have been interbred in captivity (Gier 1968; Silver and Silver 1969; Mengel 1971); direct mating between dogs (i.e., domesticated gray wolves) and coyotes in the wild may be facilitated because they are not ecological competitors and because of similarities in size and appearance of specific dog breeds with respect to coyotes. We suggest that the Y-haplotypes shared between coyotes and dogs were introgressed into coyotes from dogs, not wolves. Genetic profiling of historical samples may or may not reveal Y-haplotype sharing trends not observed in contemporary samples and analysis of additional markers could resolve differences between apparently shared Y-haplotypes (e.g., Brown et al. 2011), providing either support for our hypothesis or alternative hypotheses.

### Temporal and spatial dynamics of introgression

Considering the contrasting levels of admixture detected based on the autosomal and Y-chromosome markers analyzed in this study, we suggest the present diversity of introgressed dog genes in the greater eastern coyote population originated predominantly from historical rather than contemporary hybridization between these species. This is supported by our observation that 19% (*n* = 31 of 166) of male coyotes with *Q*_COY_ ≥ 0.8 exhibited a dog Y-haplotype (Table S2), but only 3% of coyotes exhibited autosomal admixture from dogs (*Q*_DOG_ > 0.2, Fig. 2). Specifically, 6% (*n* = 3 of 54) and 25% (*n* = 28 of 112) of historical and contemporary male coyotes with *Q*_COY_ ≥ 0.8, respectively, exhibited a dog Y-haplotype (Table S2). This indicates that the frequency of introgressed dog Y-chromosomes in coyotes in southeastern Ontario increased between 1974–1984 and 2005–2010 despite no apparent change in autosomal admixture from dogs (Fig. 2). Notably, five of the six male coyotes with *Q*_DOG_ > 0.2 exhibited a dog Y-haplotype (Table S2: *n* = 1 historical; *n* = 4 contemporary); these individuals putatively represent F1 hybrids or backcrosses. The dog admixture detected in northeastern coyotes based on SNPs is estimated to have originated ∼30 years ago (vonHoldt et al. 2011), but our Y-chromosome data demonstrated dog introgression in highly assigned coyotes sampled from southeastern Ontario >30 years ago (Table S2), indicating that hybridization had occurred earlier. This discrepancy may reflect geographical differences in sampling of eastern coyotes between studies and the likelihood that coyotes continued to interbreed with dogs at the periphery of their range as they expanded eastward (Hilton 1978; Parker 1995). We suggest that the introgressed dog Y-haplotypes observed in eastern coyotes originated from direct coyote–dog hybridization that occurred primarily historically, and concomitantly with wolf–coyote hybridization in southeastern Ontario, as coyotes expanded eastward during the last century, although coyote–dog hybridization may also have occurred during eastward expansion across the western Great Lakes states. Colonizing coyotes expanding at the eastern periphery of the species’ range would presumably have occurred at relatively low densities and may have been subject to an Allee effect (Allee 1931), encountering free-roaming dogs near human settlements in greater frequency than conspecifics, resulting in hybridization. This scenario has been documented for wolf–dog hybridization, whereby hybridization occurred in areas of low wolf density (Andersone et al. 2002; Muñoz-Fuentes et al. 2010) or was restricted to peripheral and recently expanded wolf populations (Godinho et al. 2011). The nonrecombining dog Y-chromosomes were probably introgressed into the expanding coyote population at low levels and then amplified in frequency by logistic demographic growth that presumably occurred as coyote density increased and conspecific breeding predominated (Currat et al. 2008); this could explain the common occurrence of dog Y-haplotypes in contemporary eastern coyotes despite minimal evidence of autosomal admixture from dogs. The eastern coyote may exhibit varying levels of contemporary autosomal admixture from dogs across its range.

Introgressed dog genes are common in coyotes in northeastern North America and those occurring east of the Mississippi River and south of the Great Lakes (Table 2; vonHoldt et al. 2011; Wilson et al. 2012), which may reflect the aforementioned susceptibility of an expanding population to hybridization. Morphological evidence of coyote–dog hybridization in western North America has been reported (Bee and Hall 1951; Mahan et al. 1978; Freeman and Shaw 1979), but available genetic data do not support the introgression of dog genes into coyotes in that region (Table 2; vonHoldt et al. 2011), suggesting that backcrossing of such hybrids with coyotes has been rare there. The dog (and wolf) Y-haplotypes observed in Great Lakes coyotes could be the result of in situ hybridization or gene flow from westward dispersing eastern coyotes. Additional Y-chromosome profiling could further elucidate the geographic extent of coyotes that exhibit introgressed dog Y-haplotypes.

### Conditions facilitating introgression

Coyotes and dogs interbred in captivity can produce fertile offspring (Gier 1968; Silver and Silver 1969; Mengel 1971), and wild coyote–dog hybrids have been reported (Gipson et al. 1974; Mahan et al. 1978; Freeman and Shaw 1979). However, it has been postulated that dog genes were unlikely to become introgressed into the coyote gene pool because a phase shift in the breeding season of coyote–dog hybrids makes backcrossing with coyotes unlikely (Gier 1968; Silver and Silver 1969; Mengel 1971). Additionally, it has been suggested that lack of parental care from male dogs or coyote–dog hybrids would reduce hybrid offspring survival (Silver and Silver 1969; Mengel 1971). Our findings indicate that despite these potential inhibiting factors, some hybrid male offspring from female coyote × male dog crosses must have successfully backcrossed with coyotes, as noted for offspring from a female dog × male coyote crossing (Adams et al. 2003). The backcrossing of wolf–dog hybrids with wolves has also been reported (Godinho et al. 2011). The introgression of dog Y-chromosomes into coyotes may have been possible because of variability in the breeding season of male coyote–dog hybrids that could have facilitated backcrossing with female coyotes (Gipson et al. 1975). Furthermore, pack associates may have helped provision for hybrid offspring (see Andelt 1985) in the absence of male parental care, which does not preclude mateless female coyotes potentially raising pups to independence (Sacks and Neale 2001).

### Causes of asymmetric introgression

The predominantly asymmetric nature of coyote–dog hybridization parallels that of wolf–dog hybridization, whereby hybridization typically involves a female of the wild species and a male of the domestic species (see Godinho et al. 2011). Evidence of female dog × male wolf or coyote crossings is relatively rare (Adams et al. 2003; Muñoz-Fuentes et al. 2010; Hindrikson et al. 2012), which could reflect a lack of recruitment of such hybrid offspring into wild populations. Female dogs, excepting feral ones, are unlikely to give birth in the wild, therefore their hybrid offspring would be unlikely to backcross with the wild species and presumably would not be sampled in wildlife genetic surveys (Hindrikson et al. 2012). However, the different breeding cycles of male and female dogs may facilitate asymmetric hybridization between wild canids and dogs, considering that male dogs can breed year round and therefore are capable of mating with female wild canids in heat, but female dogs only come into heat twice a year in variable seasons and therefore only a fraction of them are capable of mating with reproductively active male wild canids (Mengel 1971). Asymmetric introgression from dogs into coyotes may result partly from the potentially reduced fecundity of female coyote–dog hybrids (Mengel 1971; Gipson et al. 1975) and the potentially decreased fitness of coyote–dog hybrids with dog mtDNA relative to those with coyote (or eastern wolf) mtDNA. Notably, Haldane's Rule (Haldane 1922) does not apply for coyote–dog hybrids.

### Diagnostic value of locus MS41A

The value of locus MS41A as a diagnostic marker for distinguishing Y-haplotypes of gray wolf (and dog) origin from those of coyote (and eastern wolf) origin has been alluded to previously (Hailer and Leonard 2008), and recently *Zfy* intron sequences have confirmed species-specific origins of Y-haplotypes (Wilson et al. 2012). Based on our findings (Table 2) and those of Wilson et al. (2012), Y-haplotypes with allele 208 at locus MS41A are associated exclusively^3^ with Yint-2 of gray wolf or dog origin, and those with alleles 212, 214, or 216 at locus MS41A are associated with Yint-1 (alleles 212, 214, and 216) or Yint-3 of coyote origin (alleles 212 and 214), or Yint-4 of eastern wolf origin (allele 212). Additionally, a rare Y-haplotype with allele 196 at locus MS41A, which was observed exclusively in dogs, was associated with Yint-2 (Table 1). The *Zfy* intron(s) associated with Y-haplotypes with allele 210 or 218 at locus MS41A remain to be determined. Analysis of additional Y-chromosome SNPs is required for potentially differentiating Y-haplotypes that are apparently shared between gray wolves and dogs (e.g., Brown et al. 2011) because the *Zfy* intron fragment we sequenced lacks resolution in this respect.

## Conclusions

Our investigation of the canine Y-chromosome has further clarified the evolutionary history of the eastern coyote. Specifically, Y-haplotypes observed in eastern coyotes that were previously attributed to gray wolf introgression have now been predominantly attributed to dog introgression, although some gray wolf introgression is still evident (Table 2). We observed three incomplete Y-chromosome genotypes in coyotes in this study that appeared to be of gray wolf origin (Table S2), therefore we potentially underestimated the level of gray wolf introgression in eastern coyotes. Although allele sizes from Koblmüller et al. (2009) were unavailable for comparison, we question whether the eight “wolf clade” Y-haplotypes observed exclusively in eastern coyotes in that study were of wolf origin; we suggest the possibility that those Y-haplotypes were of dog origin.

The evidence presented herein of introgressed dog and wolf genes in eastern coyotes is corroborated by investigations of skull morphology (Lawrence and Bossert 1969; McGinnis 1979; Kays et al. 2010) and coat color (Anderson et al. 2009; Brockerville et al. 2013). The introgression of wolf genes has contributed to the large body size and skull dimensions of eastern coyotes, putatively making them more effective predators of deer (Kays et al. 2010), but the effect, if any, of introgressed dog genes on the evolution of eastern coyotes remains unknown. We speculate that behavioral traits of dogs would likely be selected against in coyotes because loss of wildness would presumably lead to increased mortality risk from humans. We are not aware of any evidence to suggest that the introgression of wolf or dog genes has resulted in more aggressive behavior of eastern coyotes toward humans.

This study and others (e.g., Iacolina et al. 2010; Wheeldon et al. 2012) highlight the importance of using multiple genetic markers when assessing hybridization between species. The standard approach for studies investigating hybridization should involve the assessment of maternally, paternally, and biparentally inherited genetic markers, and if possible should incorporate morphological data (e.g., Benson et al. 2012; Wheeldon and Patterson 2012).

## Notes

1 The published allele sizes and frequencies of multiple Y-haplotypes were erroneous; we obtained the corrected allele sizes and frequencies from the authors.

2 Male individual omitted for Y-haplotype frequency determination in coyotes.

3 A Y-haplotype with allele 208 at locus MS41A observed in a Texas coyote was associated with Yint-3 and was suggested to result from a rare homoplasy (Wilson et al. 2012).

## Data Accessibility

DNA sequences: Genbank accessions FJ687618–FJ687620 (*Zfy* intron) and Table S3 (mtDNA). Microsatellite data deposited in the Dryad Repository: http://dx.doi.org/10.5061/dryad.72s5k.

## Supporting Information

**Figure S1.** Plots of quantitative *K*-determination criteria, ln *P*(*D*) and Δ*K*, for *K* = 1–7 Structure analyses of contemporary (2005–2010) coyotes from southeastern Ontario, eastern wolves from Algonquin Provincial Park, gray wolves from northeastern Ontario, and dogs genotyped at 12 autosomal microsatellite loci. The F-model and I-model were implemented separately.

**Figure S2.** Factorial correspondence analysis of historical (1974–1984) and contemporary (2005–2010) coyotes from southeastern Ontario, eastern wolves from Algonquin Provincial Park, gray wolves from northeastern Ontario, and dogs genotyped at 12 autosomal microsatellite loci.

**Figure S3.** Structure assignments of contemporary (a = F-model; b = I-model) and historical (c = F-model; d = I-model) coyotes from southeastern Ontario, eastern wolves from Algonquin Provincial Park, gray wolves from northeastern Ontario, and dogs genotyped at 12 autosomal microsatellite loci. Each partitioned vertical bar represents an individual's proportional membership to the *K* = 4 populations analyzed in Structure.

**Figure S4.** Median-joining network of Y-chromosome haplotypes observed in coyotes, wolves, and dogs. Haplotypes are based on Y-chromosome microsatellite genotypes and are represented by variably colored nodes: WC, yellow; GLC, green; EC, orange; EW, red; GLW, purple; NA GW, blue; EA GW, gray; and DD, black (abbreviations defined in Table 2). Node sizes are not proportional to haplotype frequency. Median vectors are represented by white nodes. Branch lengths are not proportional to the number of mutational steps between nodes; branch lengths represent one microsatellite repeat (R), except where indicated otherwise.

**Table S1.** Y-chromosome haplotype identifiers provided in different studies: ID1, Rutledge et al. 2010; Wheeldon et al. 2010b; Wheeldon and Patterson 2012; and Wilson et al. 2012; ID2, Hailer and Leonard 2008; ID3, Fain et al. 2010; ID4, Sundqvist et al. 2001; ID5, Sundqvist et al. 2006.

**Table S2.** Sample information, genetic data, and Structure assignments (*K* = 4) and associated 90% probability intervals (PI) of historical (1974–1984) and contemporary (2005–2010) coyotes from southeastern Ontario genotyped at 12 autosomal microsatellite loci. Structure *Q*-values: red, averaged; yellow, F-model; blue, I-model. Abbreviations: COY, coyote; EW, eastern wolf; GW, gray wolf.

**Table S3.** Frequencies and Genbank accessions of mitochondrial DNA haplotypes observed in historical (1974–1984) and contemporary (2005–2010) coyotes from southeastern Ontario and dogs.
