# Supplementary figures and images for: Y-chromosome evidence supports asymmetric dog introgression into eastern coyotes
Source: Ecol Evol. 2013 Jul 31;3(9):3005–20. doi: 10.1002/ece3.693 (PMC3790547; doi:10.1002/ece3.693)

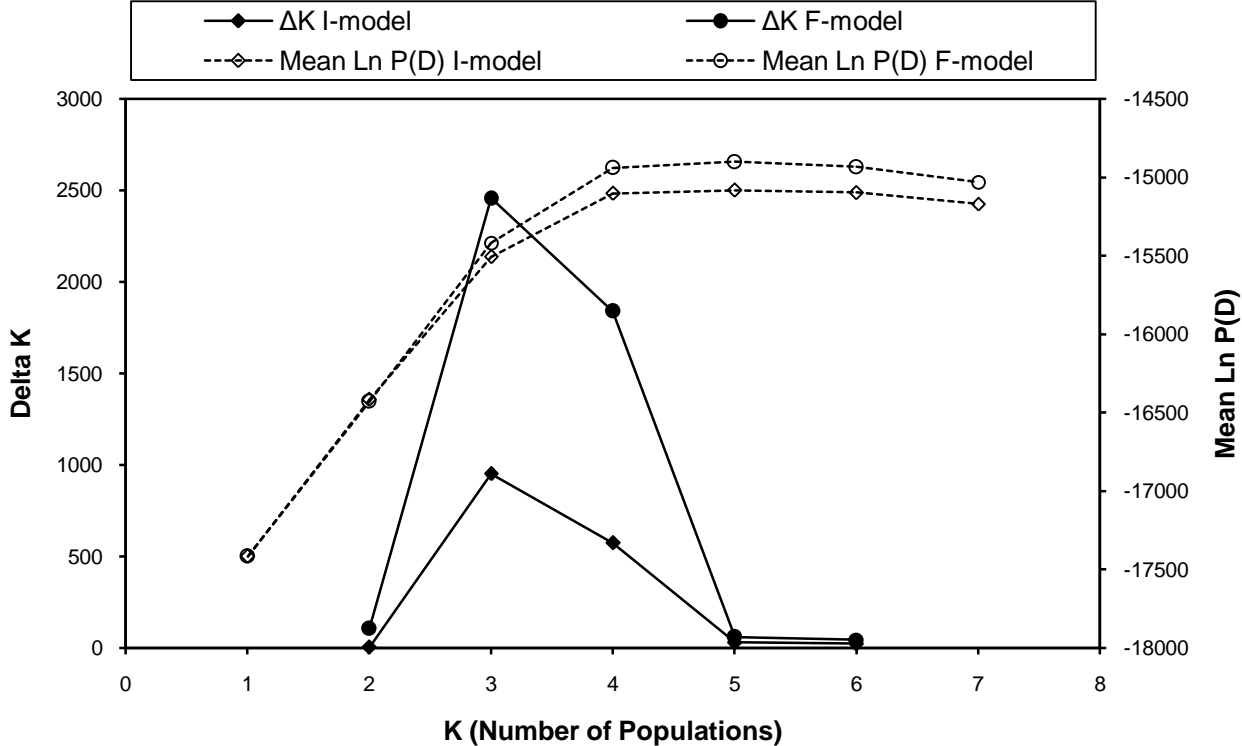

Supplement: Supplementary file 1 [file ece30003-3005-SD1.pdf]

● Coyotes 1974-1984

○ Coyotes 2005-2010

◆ Eastern Wolves

◇ Gray Wolves

▲ Dogs

FC-2 (4.73%)

FC-1 (5.32%)

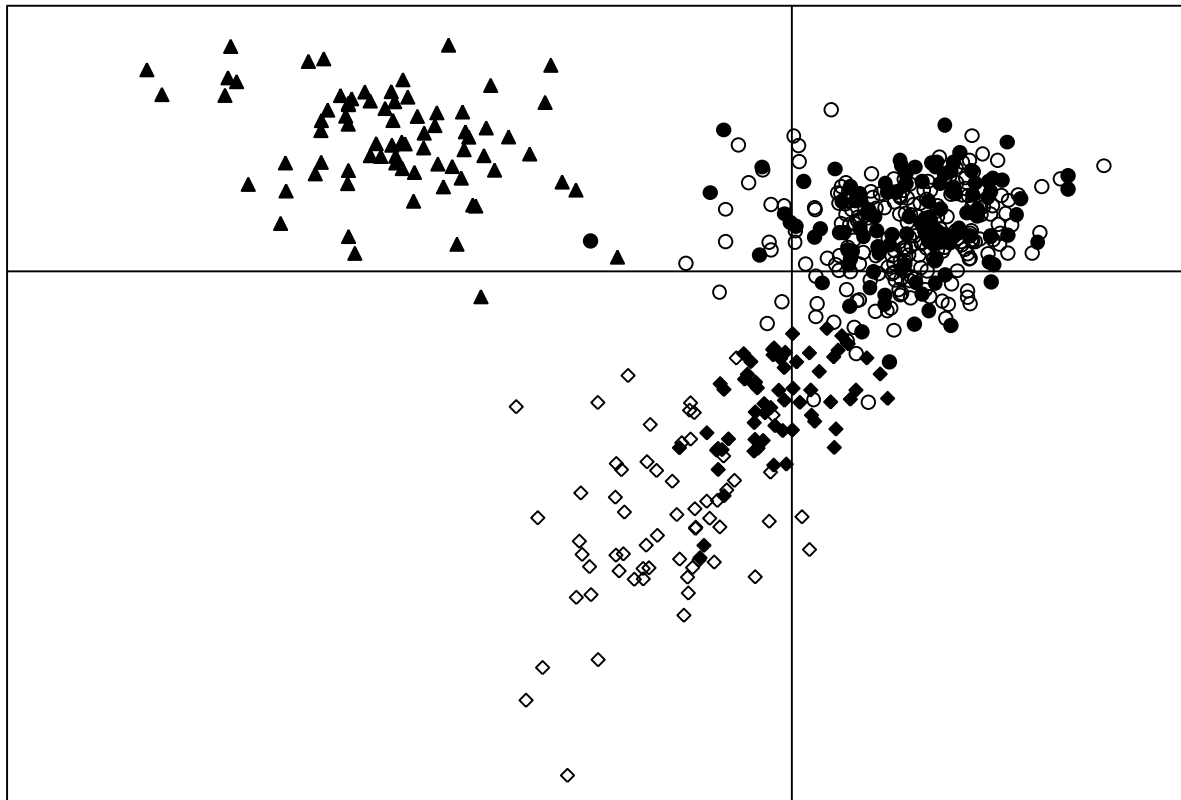

Supplement: Supplementary file 2 [file ece30003-3005-SD2.pdf]

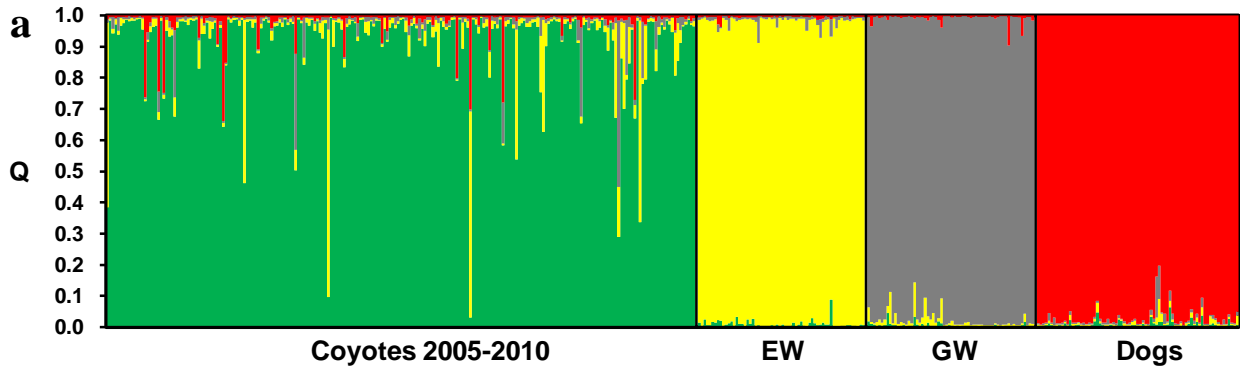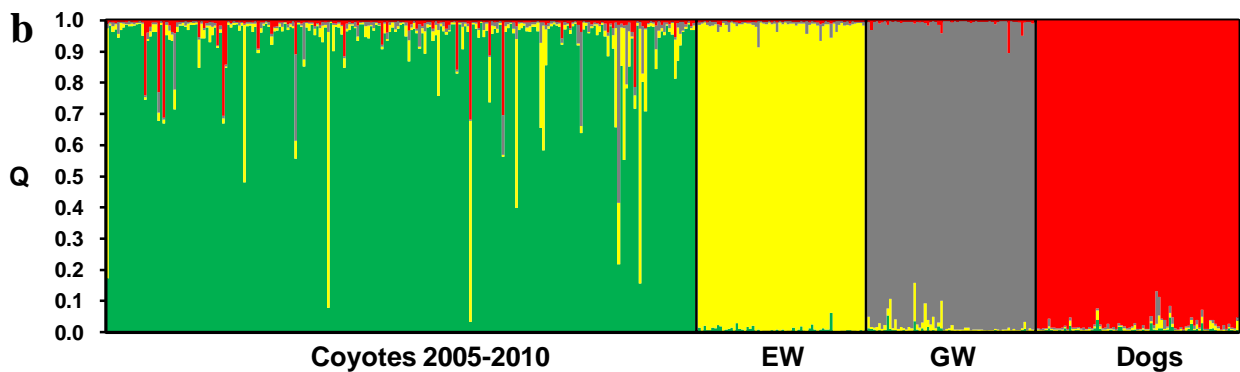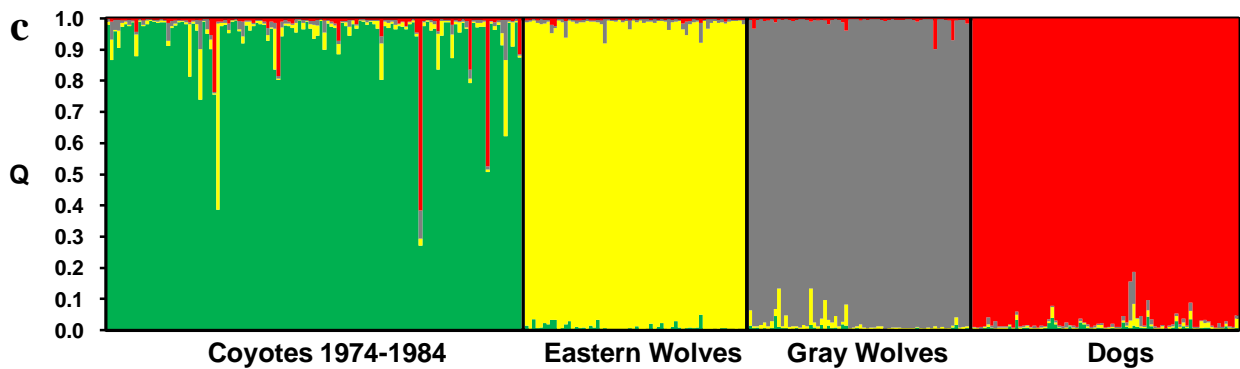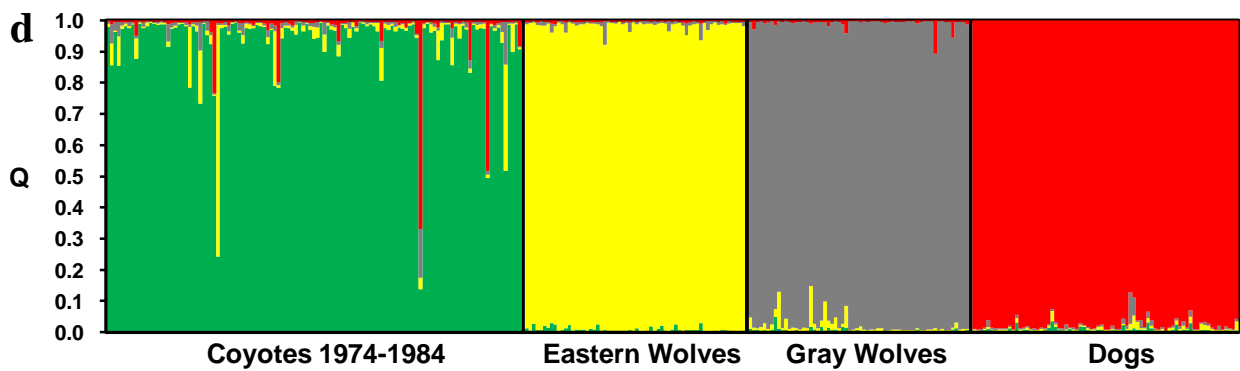

Supplement: Supplementary file 3 [file ece30003-3005-SD3.pdf]

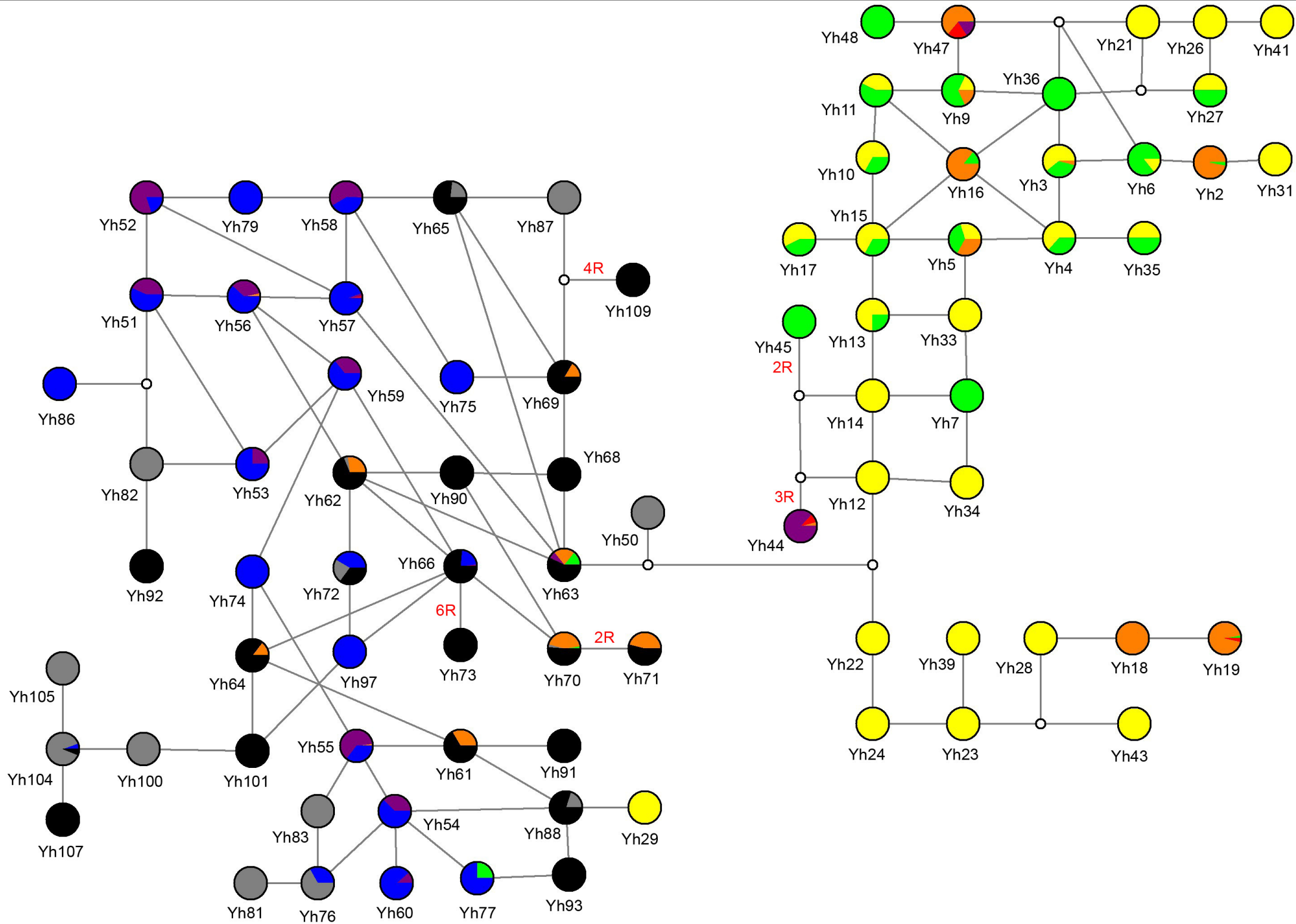

Supplement: Supplementary file 4 [file ece30003-3005-SD4.pdf]
